# Novel Photocatalytic PVDF/Nano-TiO_2_ Hollow Fibers for Environmental Remediation

**DOI:** 10.3390/polym10101134

**Published:** 2018-10-12

**Authors:** Francesco Galiano, Xue Song, Tiziana Marino, Marcel Boerrigter, Omar Saoncella, Silvia Simone, Mirko Faccini, Christiane Chaumette, Enrico Drioli, Alberto Figoli

**Affiliations:** 1 Institute on Membrane Technology, ITM-CNR, Via P. Bucci 17/c, 87036 Rende (CS), Italy; f.galiano@itm.cnr.it (F.G.); t.marino@itm.cnr.it (T.M.); omersnc@gmail.com (O.S.); s.simone@itm.cnr.it (S.S.); e.drioli@itm.cnr.it (E.D.); 2 Fraunhofer IGB, Nobelstrasse 12, 70569 Stuttgart, Germany; song.xue@outlook.com (X.S.); christiane.chaumette@igb.fraunhofer.de (C.C.); 3 LEITAT, C/de la Innovació, 2 08225 Terrassa (Barcelona), Spain; mboerrigter@leitat.org (M.B.); mfaccini@leitat.org (M.F.)

**Keywords:** hollow fiber membranes, PVDF, TiO_2_, mixed matrix membrane, photocatalytic degradation, ultrafiltration

## Abstract

Polyvinylidene difluoride (PVDF) mixed matrix membranes loaded with inorganic TiO_2_ nanoparticles have received increasing attention in the last few years as self-cleaning membranes for possible application in wastewater treatment and seawater filtration. These novel membranes show increased hydrophilicity, stability and catalytic activity under UV-A irradiation. In this work, PVDF-TiO_2_ hollow fibers were prepared by employing new strategies for enhancing the stability of the TiO_2_ dispersion, reducing particle agglomeration and improving their distribution. The spinning conditions for producing ultrafiltration hollow fiber membranes from PVDF material and nano-TiO_2_ were investigated. Finally, the optimized fibers have been characterized and tested for methylene blue (MB) degradation in water and salty seawater, revealing good permeability, long-term stability under UV-A irradiation, and photo-catalytic activity in both test solutions.

## 1. Introduction

Population growth, agricultural demand and expansion of industrialization represent the major causes of detrimental surface and underground water pollution, making it necessary at the same time to have more and more stringent environmental regulations [1]. Improvement of water quality could be guaranteed by fighting on two fronts: on the one hand, accurately monitoring water quality and on the other hand, developing highly efficient technologies for wastewater treatment. Membrane-based purification processes are among the most advanced and versatile technologies for wastewater treatment, the production of potable and ultrapure water, water recycling and desalination [2,3]. Moreover, membranes find application in industrial treatment of secondary and tertiary municipal wastewater and oil field produced water [4]. In order to increase water purity and quality, different membrane processes, complementary to each other, can be combined in many cases, thus improving the overall efficiency of water treatment [5,6]. Polymeric materials are the leading choice for membrane preparation and dominate the market due to their availability and ease of processing compared to inorganic materials. However, membrane scientists agree that the major drawbacks connected to the use of such materials are their lower mechanical resistance and intrinsic hydrophobicity. In particular, the latter reduces their resistance to fouling and biofouling, which is today defined as the “Achilles heel” of membrane processes [7]. Several strategies have been proposed and tested for improving the hydrophilicity of polymeric membranes; each of them has advantages and drawbacks. Among them, the preparation of mixed matrix membranes (MMMs), incorporating inorganic nanoscale materials, seems to be one of the most promising [8,9]. Titanium oxide (TiO_2_), having excellent chemical stability, low cost and non-toxicity, is one of the most investigated materials for this purpose [10,11,12,13]. Moreover, besides improving membrane hydrophilicity, TiO_2_ offers the possibility of combining the filtration process with photocatalysis. This synergy generates a powerful system, which can be exploited for the contemporaneous separation and degradation of harmful pollutants [8,14,15,16], one-step synthesis of organic compounds [15], and hydrogen generation from water splitting [15]. PVDF is among the most used polymers for membrane preparation due to its outstanding properties; however, its intrinsic hydrophobicity is a major obstacle to its full application in water and wastewater treatment [17]. Several works have investigated the possibility of combining PVDF membranes with TiO_2_ nanoparticles (NPs). A survey of recent and relevant publications is presented in Table 1.

It can be seen that several works use commercial TiO_2_ [18,22,33], while other authors performed NPs synthesis via sol/gel methods starting from TBOT or titanium (IV) iso-propoxide (TTIP) [19,21]. Table 1 is divided into two sections, depending on the method selected for preparing PVDF/TiO_2_ membranes. In fact, there are two strategies for preparing such membranes, and each author claims its advantages. While coating seems to offer improved photo-catalytic activity, due to better accessibility to the catalyst particles their incorporation in the polymeric matrix is easier, can be done in one step and reduces the risk of their loss in the permeate. 

For both strategies, agglomeration of the NPs remains one of the main obstacles for generating a uniform surface coating or even distribution in the membrane matrix to achieve improved fouling resistance. When incorporated in the membrane, agglomerations can provoke pore plugging or induced defects in the selective top layer. Additionally, the agglomeration of TiO_2_ reduces the specific surface area, and therefore, the photocatalytic degradation capability. For achieving a uniform coating layer or good distribution in the membrane matrix, the degree of particle agglomeration should be minimized. Generally, it is not enough to break NPs apart as in colloidal systems re-agglomeration takes place due to the Van der Waals forces between NPs. The re-agglomeration can be mitigated by introducing charges (electrostatic stabilization) and physical barriers (steric stabilization) on the surface of TiO_2_ NPs. In colloidal systems, electrostatic stabilization can be achieved by the addition of charges on the surface of the NPs so that they can repel one another at non-isoelectric pH. Steric stabilization is achieved by adsorbing surfactants, polyelectrolytes, polymers or other modifiers onto the nanoparticle surface to physically prevent the NPs from coming close enough to each other to cause agglomeration [41]. Razmjou et al. used combinations of mechanical and chemical modification approaches for reducing particle agglomeration, achieving a significant improvement in fouling behavior and hydrophilicity. For the chemical modifications silane coupling agents were used [12]. Other researchers effectively modified the properties of colloidal TiO_2_ suspensions by introducing surfactants, which adsorb at the solid–liquid interface [42,43] while Liufu et al. used the adsorption of polyelectrolytes on the TiO_2_ NPs for stabilizing the aqueous suspensions [44]. Besides improving the dispersion stability of NPs, the addition of surfactants like sodium dodecyl sulphate (SDS) can also influence the membrane morphology and structure and increase the membrane hydrophilicity [45]. Researchers reported improved membrane surface properties, enhanced hydrophilicity and anti-fouling properties for the TiO_2_ modified membranes. In some papers, the photocatalytic activity of the produced membranes was also tested in order to demonstrate their potential applications [20,27]. Conventional sonication and grinding can be used to modify the surface of TiO_2_ NPs [12]. 

Chemical modifications, mainly based on the use of aminopropyltriethoxysilane [12] and γ-amminopropyltriethoxysilane [46], were reported as efficient methods for improving titania dispersion in the polymeric solution. The possibility of increasing hydroxyl groups on the membranes surface, thus promoting polymer-semiconductor strong interactions, has also been investigated [47,48]. 

In this work, PVDF hollow fiber membranes loaded with TiO_2_ NPs were produced. In the first part of our research, the dope solution composition was optimized, starting from our previous works on PVDF hollow fiber preparation [49,50]. Then, new strategies for enhancing the stability of the TiO_2_ dispersion, reducing particle agglomeration and improving distribution were studied. In particular, chemical modification with SDS was combined with mechanical modification using ball milling and ultrasonics to improve the TiO_2_ dispersion and distribution in the membrane matrix. One of the strategies considered was the concomitant addition of PEG, which further promoted the dispersion of the TiO_2_ NPs preventing their agglomeration. Finally, the optimized fibers were characterized and tested for MB degradation in water and synthetic seawater, revealing good permeability, long-term stability under UV-light, and excellent photo-catalytic activity without chloride poisoning.

## 2. Experimental

### 2.1. Materials

PVDF Solef^®^6012 polymer powder was kindly provided by Solvay Specialty Polymer (Bollate, Italy). Polyvinylpyrrolidone (PVP) (Luvitec^®^ K17) was purchased from BASF (Treviso, Italy) and dried at 60 °C overnight prior to use. Poly(ethylene glycol) 400 (PEG-400) and titanium(IV) oxide nanopowder (P25 Degussa) were purchased from Sigma-Aldrich (Milano, Italy) and used without further purification. Sodium dodecyl sulphate (SDS) and perfluoro-compound FC-40^TM^ (Fluorinert^TM^ FC-40) were purchased from Fisher Scientific (Milano, Italy). *N*-Methyl-2-pyrrolidone (NMP) (Carlo Erba) was used as a solvent without further purification. Sodium hypochlorite (NaClO), glycerol and ethanol were purchased from Carlo Erba, Milano, Italy and used diluted to remove residual PVP, or as pore-preservation agent for membranes post-treatment. In all cases, tap water was used as the external coagulation bath in the spinning process.

MB (Reag. Ph. Eur. Grade) powder was purchased from Merck (Darmstadt, Germany).

Deionized water was obtained from a water purification system (Zeneer RO 180, Human Corporation, Seoul, Korea), a Hermle Z36HK centrifuge was used to recover functionalized NPs.

### 2.2. Nano-TiO_2_ Dispersion

Two different approaches combining chemical modification with mechanical modification were investigated to improve the stability and the dispersion of TiO_2_ NPs in the dope solution.

In the first approach, P25 Degussa TiO_2_ NPs were first chemically functionalized with anionic surfactant SDS in aqueous solution. Therefore, 1.4 g of SDS was dissolved in 200 mL of distilled water and successively 1g of TiO_2_ NPs was slowly added. The white suspension obtained was stirred for 6 h after the functionalized TiO_2_-SDS NPs were recovered by centrifugation at 6000 rpm for 10 min and finally dried overnight at 45 °C in an oven. Functionalized NPs were then mechanically dispersed by stirring (30 min) and sonication (30 min) in the amount of H_2_O required for the dope solution preparation.

In the second approach, the TiO_2_ NPs were first chemically modified as described above, which was subsequently followed by mechanical modification in the organic solvent solution. In particular, the dried chemical functionalized TiO_2_ NPs were then manually ground to a fine powder by using a mortar. The modified TiO_2_ was suspended in NMP solvent under continuous stirring until a stable dispersion was obtained. Subsequently, the amount of PEG 400 (required for the dope solution preparation) was added and the suspension was stirred for 1 h. Thereafter the suspension was sonicated using a Sonics Vibracell VCX 750 (Sonics, Newtown, CT, USA) using 40% of amplitude maintaining the temperature during sonication below 30 °C. 

### 2.3. Nano-TiO_2_ Particle and Dispersion Characterization

The TiO_2_ particle size distribution and zeta potential of the aqueous suspensions was characterized at 25 °C using a particle size analyzer Zetasizer Nano ZS from Malvern Instruments Ltd. (Malvern, UK). The measuring principles are based on analyzing the dynamic fluctuations of light scattering intensity caused by the Brownian motion of the particles. The hydrodynamic radius, or diameter, was calculated via the Stokes-Einstein equation from the aforementioned measurements. The potential stability of the suspensions was studied by measuring the electrophoretic mobility using a zeta potential analyzer Zetasizer Nano ZS from Malvern Instruments Ltd. (Malvern, UK). The particle size distribution and electrophoretic mobility measurements were studied by using small amounts of modified and unmodified TiO_2_ in aqueous solutions (0.15 wt %). Moreover, a visual stability test in organic solvents was performed to determine the ability of the TiO_2_ nanoparticle suspensions to remain dispersed over a period of time. Photos of the suspensions prepared using the various dope compositions inside clear glass containers were captured at certain intervals. 

### 2.4. Hollow Fibers (HFs) Preparation

The appropriate amount of solvent was heated at 80 °C in a 2 neck round bottom flask using an oil bath. The additives (PVP k17, PEG400) and polymer (PVDF 6012) were slowly added into the pre-heated solvent using mechanical stirring. The TiO_2_ NPs (prepared with the two approaches described above) were then added as a water formulation (HF 1-0.5) or a PEG 400 formulation (HF 2-0.5). The compositions of dope solutions used in this study is presented in Table 2. The HFs containing the TiO_2_ produced using the first approach for dispersing the TiO_2_ powder are named HF 1-0.5, while the HFs following the second approach are named HF 2-0.5. Moreover, PVDF HFs without TiO_2_ were also prepared, as reference samples, and labelled as HF 1-0 and HF 2-0, respectively.

Dope solution was kept at a selected temperature overnight and then transferred to a heated tank and spun through a spinneret. The dry/wet spinning technique was used for HF fabrication, as described elsewhere [49,50]. The detailed conditions of fiber spinning (such as bore fluid composition and flowrate, coagulation bath composition, spinning rate) are reported in Table 3.

The produced HFs were washed in hot water (60 °C) and then treated overnight in a sodium hypochlorite (NaClO 4 g/L) solution for PVP removal as described in previous works [49,50]. In order to avoid collapse of the fibers’ porous structure, they were soaked for 4 h in a glycerol aqueous solution, with concentration of 30 wt %, before drying, as already described elsewhere [49,50]. Before any permeability test, the glycerol was completely washed out with hot deionized water (60 °C).

### 2.5. Characterization of HF Membranes

#### 2.5.1. HFs Morphology and Elemental Analyses

The morphology of the PVDF HFs was observed by Scanning Electron Microscopy (SEM) (Quanta FENG 200, FEI Co., Hillsboro, OR, USA). Fibers were freeze fractured using liquid nitrogen to analyze their cross-sectional morphology. A thin conductive gold layer was coated on pure PVDF HFs to improve the imaging resolution and to prevent electrical charging, while a carbon layer was coated on TiO_2_-doped HFs to avoid interference with the inorganic material.

Energy-dispersive X-ray diffraction (EDX) and backscattered detector (BSD) measurements were carried out with an Electron Probe Micro Analyzer (EPMA)-JEOL-JXA 8230 (Jeon, Akishima, Japan). The samples were sputter coated with a thin layer of graphite prior the analyses (Carbon Coater QUORUM Q150T-ES).

#### 2.5.2. HFs Porosity, Mechanical Properties and Contact Angle

Fiber porosity, ε, was measured gravimetrically, as described in literature [49]. Fibers’ Young’s modulus and elongation at break were measured by using a ZWICK/ROELL Z 2.5 test unit as described elsewhere [51]. Briefly, each fiber type was cut (6 cm length) and stretched unidirectionally at the constant rate of 5 mm/min at room temperature until their break. From the resulting stress/strain curve the mechanical properties of the fibers were calculated by the software. For each fiber at least five measurements were carried out and the average and standard deviation were then calculated. 

Contact angle was determined by the method of the sessile drop using a CAM200 instrument (KSV Instrument LTD, Helsinki, Finland). The contact angle was determined on the outer surface of the fibers. 

#### 2.5.3. Bubble Point and Average Pore Size

Capillary flow porometer tests were carried out using the instrument, CFP 1500 AEXL (PMI porous materials Inc., Ithaca, NY, USA) for measuring the bubble point and average pore size of the fibers, as described elsewhere [52]. Perfluoro-compound FC-40^TM^ (Fluorinert^TM^ FC-40, Sigma-Aldrich, Milano, Italy) was used as a wetting liquid and tests were performed using the wet up/dry up method [53]. For each fiber at least three measurements were carried out and the average and the standard deviations were calculated.

#### 2.5.4. Stability Tests of Membranes under UV-A Irradiation

Stability of produced HFs was tested by comparing the pure water fluxes with and without UV-A-irradiation. For stability tests HF 1 membranes were selected as model fibers. Ten modules of each hollow fiber membrane type (HF 1-0 and HF 1-0.5) were prepared. Each module contained three HFs. Of the defect-free modules in wetting equilibrium, five modules were kept in the dark and five modules were irradiated.

The irradiation light sources used in this study were black light PHILIPS TL-D 18 W BLB 1SL (Philips, Amsterdam, Netherlands) with λ_max_ at 365 nm [54]. In order to irradiate multiple membrane modules simultaneously with one lamp, they were fixed in a lamp stand holding a maximum of 8 glass modules at a fixed distance of 12 cm to the UV-A lamp. In this setup the membranes in the modules were irradiated only from one side. For this reason, they were rotated by 90° every 60 min. UV-A intensity of 0.63 to 0.66 mW/cm^2^ was recorded with a YK-35UV sensor produced by Lutron Electronic behind borosilicate glass tubes and planes of the same thickness and type as the membrane modules. Internal shadowing of the membrane fibers is neglected in the evaluation.

Pure water flux of the irradiated and non-irradiated membrane samples was determined after 1, 2.5, 5, 10, 25 and 50 h of irradiation of the complete membrane surface (with double this time spent in the irradiation stand).

#### 2.5.5. Pure Water Permeability (PWP) Measurements

Water permeation experiments were conducted in a cross-flow filtration mode setup with lab-made glass modules containing 3 HFs (20 cm length) with a membrane area of 0.0036 m^2^ each. PWP tests were performed at ambient temperature in dead-end filtration mode. Deionized water was fed from a nitrogen pressurized tank with 0.5 bar (Wika Manometer 232.50.100, range 0–1.0 bar) and the permeate weight increase was recorded over 20 min using an Ohaus Explorer^®^ EX6201 scale (Ohaus, Parsippany, NJ, USA) with digital output. Due to the low permeate flow rate, no back-pressure on the permeate side of the membrane could be safely assumed when characterizing polymeric membrane samples. PWP setup for measuring the cross-flow flux in an outside-in configuration was used. The details of the setup have been already reported in [55]. For each fiber at least three measurements were carried out and the average and the standard deviations were calculated.

#### 2.5.6. Photocatalytic Activity

For the measurement of catalytic activity MB was pre-adsorbed, in accordance to what is described in ISO 10678: 2010 [56], by adding conditioning solution (20 μmol/L in deionized water) into the membrane module (around 4 L/m^2^) and storing it in the dark for 12 h before the measurement.

Catalytic activity was then measured by circulating 250 mL of 10 μmol/L MB solution in cross-flow mode over a single membrane module. Trans-membrane pressure (TMP) was kept at 0.5 bar and flow velocity was around 0.06 m/s. Both retentate and permeate were recirculated to the feed tank.

The membrane glass module containing three fibers of approximately 200 mm length and a membrane of 0.0036 m^2^ was placed in the middle of two PHILIPS TL-D 18 W BLB 1SL lamps (λ_max_ = 365 nm). The distance of the membrane to the UV lamps was 6 cm, and the estimated light intensity at the membrane surface was 2.7 mW/cm^2^ taking the glass absorption into account. Irradiation was started after 30 min of recirculation. During the overall 300 min of experiment (first 30 min without irradiation and then 270 min with UV-A irradiation), 25 samples of 1 mL were taken from the feed tank and their MB concentration was immediately determined by measuring the light absorption at 664 nm. 

## 3. Results and Discussion

### 3.1. TiO_2_ Modification

**Figure 1:**
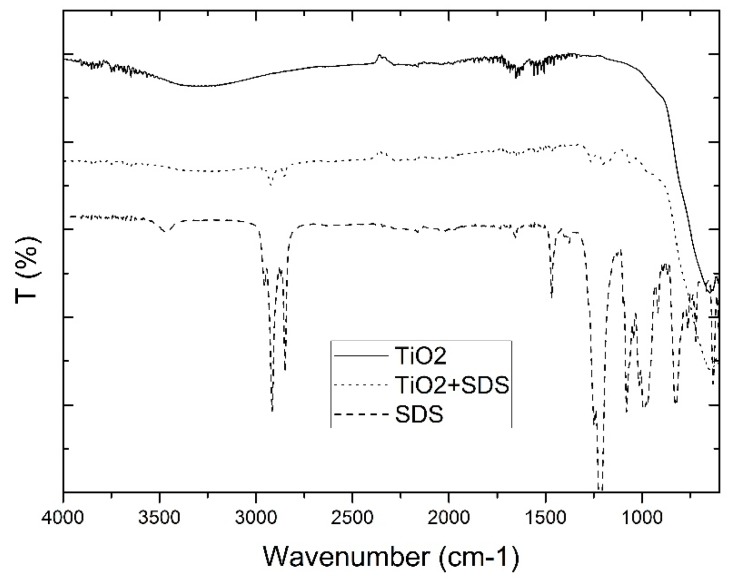
ATR spectra from sodium dodecyl sulphate (SDS) modified TiO_2_.

For the chemical SDS modified and unmodified TiO_2_ powders, the ATR spectra were measured as shown in Figure 1. In the spectra from pure SDS, small peaks between 2700 and 3000 cm^−1^ were observed as well as peaks between 1000 and 1500 cm^−1^. These peaks were also observed in the SDS modified TiO_2_ spectra whereas these peaks were not present in the unmodified TiO_2_ powder. Two strong bands appear at 2924 and 2853 cm^−1^ which correspond to the asymmetric and symmetric stretching frequencies of the CH_2_ modes of the SDS (C12) surfactant hydrocarbon tails. The band found at 1467 cm^−1^ (CH_2_ scissor mode) is common to SDS. The SO_2_ asymmetric vibrational feature ((SO_2_)) is the most intense band in the SDS spectrum. It is a combination of several overlapping peaks, and it is generally observed as a double band, located at 1219 and 1249 cm^−1^ [57]. 

**Figure 2:**
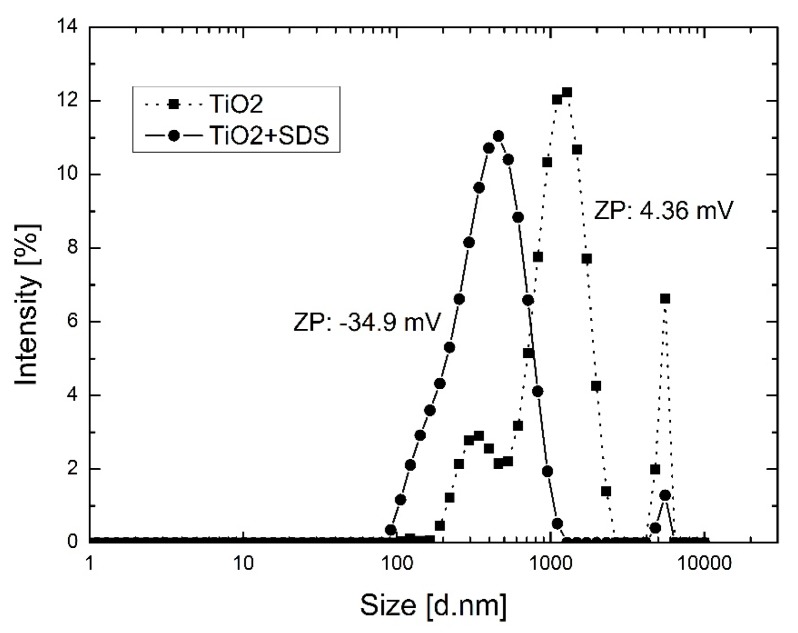
Particle size distribution and zeta potential of SDS modified and unmodified TiO_2_ NPs in aqueous solutions.

The results of the particle size distribution and zeta potential of modified and unmodified TiO_2_ in aqueous solutions is shown in Figure 2. The modification of the TiO_2_ particles with SDS shows an average particles size reduction from about 1000 nm to about 500 nm. Moreover, an increase in stability was observed for the SDS modified TiO_2_ particles, confirmed by the shift in absolute value of the zeta potential from 4.36 mV for the unmodified TiO_2_ to −34.9 mV for the SDS modified TiO_2_. In dispersions where the value of the zeta potential is close to zero, particles tend to agglomerate. At highly negative or positive values of the zeta potential (more than 30 mV or less than −30 mV) particles in dispersions tend to repel each other and no agglomeration occurs [41].

**Figure 3:**
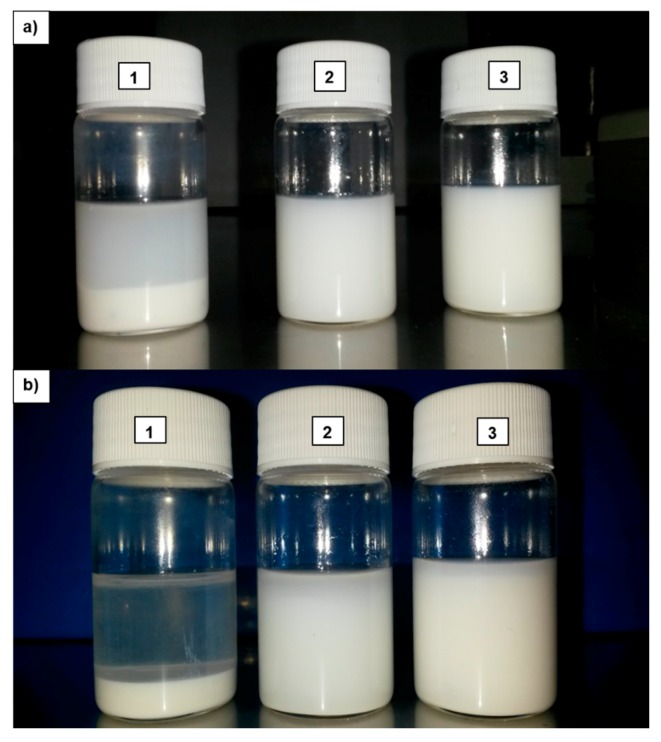
Samples dispersion in NMP of: (1) TiO_2_ P25 (not functionalized) (2) TiO_2_ chemically functionalized with SDS (HF 1-0.5), and (3) TiO_2_ (LEITAT solution) dispersed in solvent (HF 2-0.5) after 1 h (**a**) and after 24 h (**b**); respectively.

The samples of the dope suspension of TiO_2_ obtained in this work are shown in Figure 3. In particular, it can be noticed by comparing the un-functionalized NPs with modified ones, that higher stability was achieved by modification. In fact, TiO_2_ NPs are prone to precipitate very fast and so after one hour they form a white precipitate on the bottom of the flask (Figure 3, image a1), which is magnified after 24 h (Figure 3, image b1). The suspensions of chemically and mechanically modified NPs (Figure 3, image a2, a3, b2 and b3) show very similar behavior in terms of stability.

The suspension in image a3 shows better long-term stability in comparison with the suspension of image a2, which can be ascribed to the difference in dope composition. The dope composition shown in image a3 (used for preparing the membranes HF 2-0.5) contains the additive PEG which is a hydrophilic polymer and frequently used as steric stabilizer, especially in biological applications. The substitution of additive H_2_O with PEG in dope composition HF 2-0.5 resulted in additional steric stabilization, and therefore, in better long-term stability.

### 3.2. HF Characterization

#### 3.2.1. SEM and EDX

**Figure 4:**
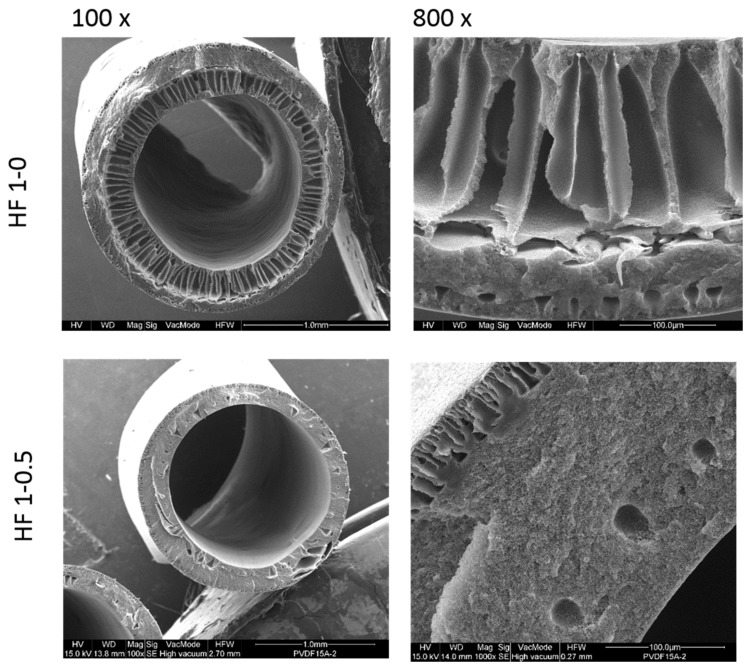
Cross-section SEM images of HF membranes at different magnitude.

The morphology of PVDF HFs 1-0 and HF 1-0.5 is shown in Figure 4.

The HFs display an asymmetric structure with open finger-like macrovoids on the inner wall, while a denser sponge-like structure with small tear-drop voids is visible on the outer surface layer of the membranes. The same morphology was already obtained in previous works [50,52] and discussed there.

As can be noticed for HF 1-0.5, the spongy morphology is predominant on the cross section probably due to a low de-mixing rate during the phase inversion process. Given that the bore fluid compositions are the same for both membranes (HF 1-0 and HF 1-0.5), this phenomenon can be ascribed to a slower kinetic in the solvent/non-solvent exchange due to strong interaction between well dispersed NPs and the polymer chains, which also led to a high dope viscosity. 

**Figure 5:**
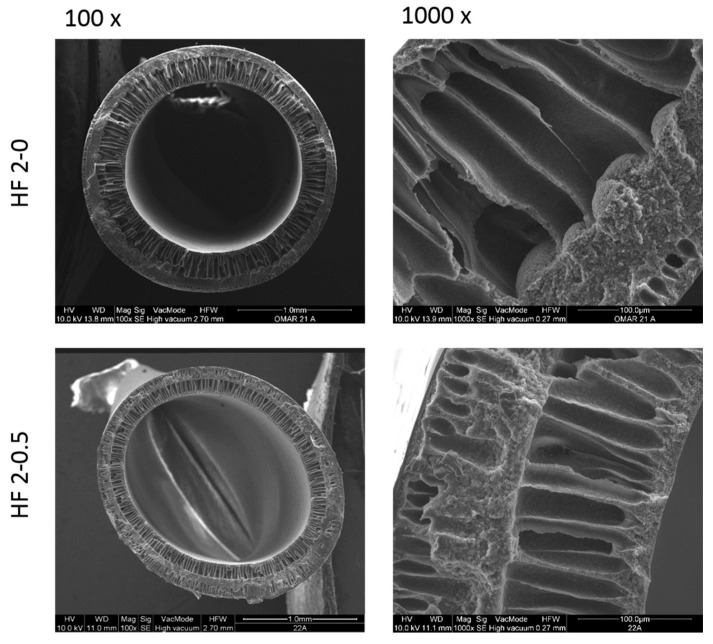
Cross section of HF 2-0 and HF 2-0.5 membranes at different magnification.

In Figure 5 the cross-section morphology of HF 2-0 and HF 2-0.5 is reported.

Pure PVDF HFs 2-0 show a sponge-like structure on the outer side and a finger-like structure on the inside of the membrane while dope solution loaded with TiO_2_ NPs (HF 2-0.5, second approach functionalization) yield a more open macrovoid structure on the outer side. This could be due to the presence of TiO_2_ which probably, thanks to its high hydrophilicity, promotes the growing of macrovoids once in contact with the water of the coagulation bath. Dope solution loaded with PEG additive led to a larger finger-like structure due to a less thermodynamically stable system that promoted rapid demixing during the phase inversion process as already reported by Wongchitphimon et al., 2011 [58].

**Figure 6:**
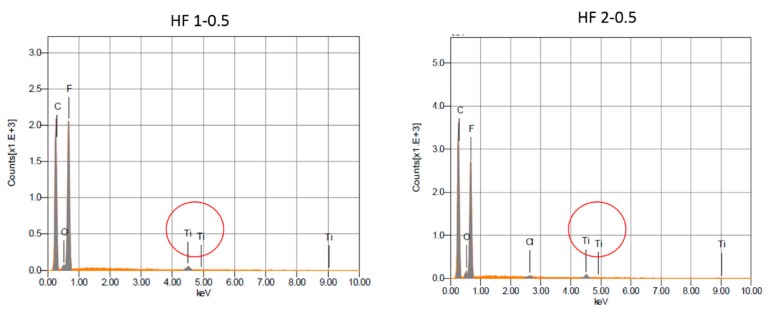
EDX analyses of HF 1-0.5 and HF 2-0.5 membranes with the peak of Ti circled in red.

**Figure 7:**
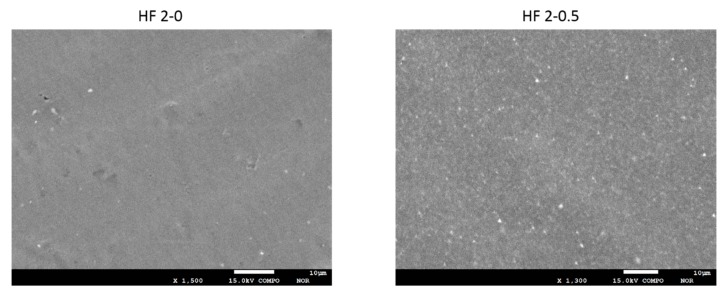
BSD images of HF 2-0 and HF 2-0.5 outer surface.

The presence and distribution of TiO_2_ NPs in the membrane matrix was assessed by means of EDX elemental analysis and BSD. The detection of the peak of Ti in HF 1-0.5 and 2-0.5 HFs was proof of the effective embodiment of the NPs into the membrane matrix (Figure 6). In Figure 7, as an example, the outer membrane surface of HF 2 type membranes, unfilled and filled with TiO_2_ NPs are compared. In HF 2-0.5 the brighter spots are clearly visible, which indicates the uniform distribution of the TiO_2_ NPs along the HF outer surface. The addition of PEG in the dope solution, in fact, prevents the agglomeration of TiO_2_ nanoparticles as also already demonstrated in other applications [59].

#### 3.2.2. Thickness, Porosity and Mechanical Properties

From the data reported in Table 4, it can be noticed that membranes prepared without TiO_2_ NPs presented similar values of thickness (HF 1-0 and HF 2-0). Similar values of thickness were also found for the membranes prepared with TiO_2_ NPs (HF1-0.5 and HF 2-0.5). It can be seen that the dimensions of the HFs (in terms of O.D. and thickness) are decreased by the addition of TiO_2_ NPs. This can be ascribed to the high forces between the long polymeric chains and the molecules on the surface of the inorganic NPs that can interact with each other, increasing the cohesive energy between polymer and inorganic NPs. The porosity of the membranes was not influenced significantly by the addition of TiO_2_ NPs.

Mechanical tests (Table 5) showed that the addition of TiO_2_ led to an increase in Young modulus, which corresponds to a lower resistance to stress deformation. For fibers obtained with the chemical functionalized TiO_2_ (HF 1-0.5), more than a two times Young modulus value was obtained (from 40.91 to 92.23 N/mm^2^), with a slight decrease of elongation ratio (from 182.59% to 167.85%). A similar trend in Young modulus was observed for HF 2 membranes which also kept good performances in terms of elongation at break. As reported in other works [60], the introduction of TiO_2_ NPs improves the mechanical resistance of the membranes by either acting as crosslinkers between the polymer chains, increasing their rigidity, and therefore, their mechanical strength [61] or through the decrease or suppression of macrovoids [62]. 

Dìez-Pascual et al. [63], for instance, commented that this improvement can be attributed to the effect of the rigid and homogeneously dispersed TiO_2_ NPs together with the interactions (H-bonding) they establish with the membrane matrix. This results in a decrease in polymer chain motion and in promoting the adhesion between the two phases.

#### 3.2.3. Pore Size

The bubble point and the pore size results of the produced membranes are summarized in Table 6.

The bubble point of the membranes is marginally influenced by the addition of TiO_2_ or by the composition of the dope solution. In fact, only a slight increase (less than 10%) of the bubble point values was observed for the HF loaded with TiO_2_ vs pure PVDF HF. All the produced HFs had a similar pore distribution with a mean pore diameter of about 0.13/0.14 µm, which places these membranes in the range of micro/ultrafiltration. 

#### 3.2.4. PWP Tests

**Figure 8:**
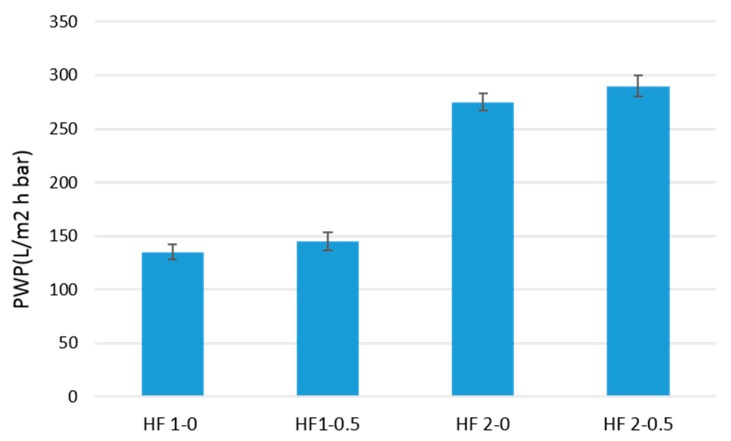
Pure water permeability (PWP) of PVDF HFs produced.

PVDF HFs and TiO_2_-PVDF were characterized in terms of PWP and the results are presented in Figure 8.

Comparing the values of PWP for the two types of HFs produced, it can be observed that HFs-2 have double the values of the PWP of the HFs-1 membrane. The use of a hydrophilic pore-former agents, such as PEG, in the dope solution can explain this large increase in membrane performance. In other words, the particular morphology of HF-2, where the wide finger macrovoids are present in the outer layer, allows lower resistance for water permeation compared to the more compact sponge like structure of the outer layer of HFs-1. For HFs-1 the PWP have technically the same value, while for HFs-2 an increase in the range of 10% was observed for HF loaded with TiO_2_. This could be due to the presence of TiO_2_ NPs, which allows higher flux through the membrane by improving its hydrophilicity. This result was confirmed by contact angle measurements carried out on both HFs-2. HF 2-0.5, loaded with TiO_2_, presented a contact angle of 78° ± 2°, while HF 2-0 presented a contact angle of 86° ± 4°. The addition of TiO_2_ in polymeric materials is very well documented to have an effect on enhancing the hydrophilicity of the membranes [64]. 

#### 3.2.5. Permeation Flux Stability under UV Irradiation

No change in membrane structure and pure water flux was observed for the PVDF HFs 1-0 membrane samples and the TiO_2_ doped PVDF membrane samples HF 1-0.5 after 50 h of 0.6 mW/cm^2^ UV-A irradiation.

The comparison showed no difference between irradiated and non-irradiated samples.

Also, no PWP increase was observed in modules irradiated during flux measurement. These results are opposite to the ones previously obtained when the TiO_2_ NPs were used for the preparation of PES catalytic HFs. Here, the membranes were demonstrated to be not resistant to the UV light exposure as evidenced by the increase in water permeability [55].

### 3.3. Photocatalytic Activity

For the photocatalytic tests the HFs-2 were selected due to their better performance in terms of water permeability.

**Figure 9:**
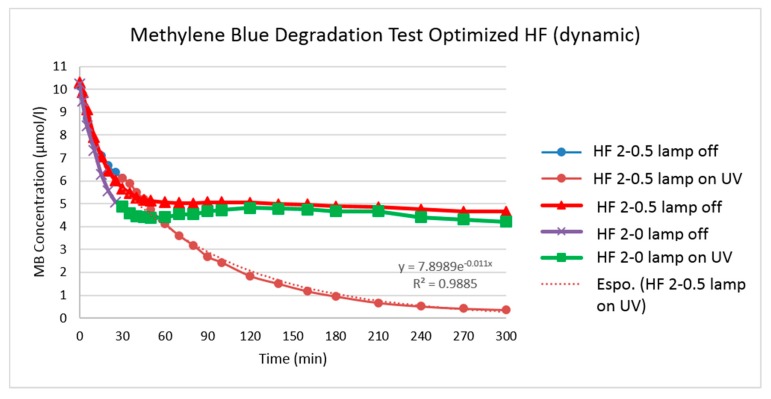
Methylene blue (MB) degradation test for HFs type 2.

The kinetics results for the MB photo-degradation tests are represented in Figure 9.

The HFs loaded with TiO_2_ (HF 2-0.5) shows a clear degradation of MB under UV irradiation (97% after 300 min). As shown in Figure 9, after the first adsorption period with the UV lamp off (30 min), the MB concentration decreased from 5.68 to 0.41 mol/L with the UV lamp on (blue and brown line). Another long-time experiment (300 min) on the same type of membrane keeping the lamp off was conducted to verify whether the blenching of MB was caused by sorption or degradation (red line). Thus, the MB concentration gap between the non-irradiated and irradiated modules is a result of photo-degradation with TiO_2_/UV association. As a reference, the same UV degradation test was conducted with HF 2-0 not loaded with TiO_2_ (violet and green line) where no degradation was observed.

In order to describe the effect of MB concentration on photo-degradation rate, the Langmuir-Hinshelwood model is widely applied (Equations (1) and (2)) [16,65,66].

(1) $$r=-\frac{dC}{dt}=\frac{kKC}{1+KC}$$

(2) $$\mathrm{after}\;\mathrm{integration}:\;ln\left(\frac{C_{0}}{C}\right)+K\left(C_{0}-C\right)=kKt=k_{app}t$$

(3) $$C_{t}=C_{0}\cdot e^{-k_{app}t}\;or\;ln\left(\frac{C_{t}}{C_{0}}\right)=-k_{app}t$$

where *r* is the oxidation rate of MB, *C*_0_ is its initial concentration, *C* is the concentration of MB after time *t* of the photocatalytic decomposition, *k* is a true rate constant, *K* is the constant of adsorption equilibrium and *k_app_* is the apparent rate constant of a pseudo first order reaction. 

When the MB solution is highly diluted (10 µmol/L), the term *KC* becomes ≪1, the denominator of (Equation (1)) can be neglected and the reaction can essentially be simplified to the apparent rate order equation (Equation (3)).

**Figure 10:**
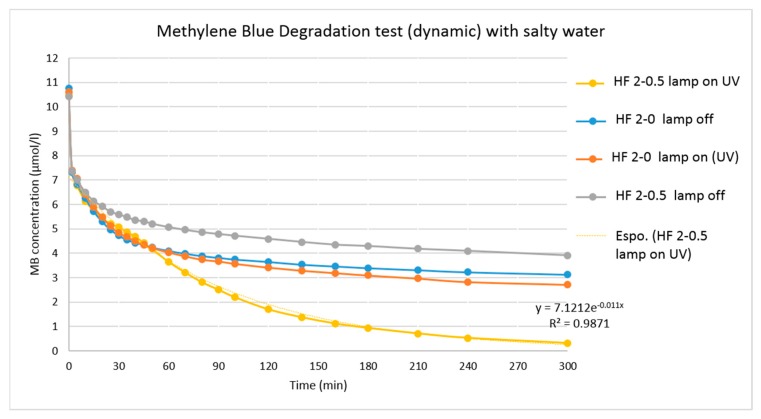
MB degradation test for HFs type 2 in 25 g/L of NaCl solution.

As observed in Figure 10, the kinetics of MB degradation on the TiO_2_ loaded membranes with UV-A showed good fit (*R*^2^ > 0.98) to pseudo first order reaction (Equation (3)) in agreement with the Langmuir-Hinshelwood model. 

A comparison of different kinds of HF degradation rates by merely looking at the reaction rate *k_app_* in the MB photo-degradation tests (dynamic) is not adequate, due to the different membrane surface area. Thus, to compare the degradation rate of different kinds of HF, the specific degradation rate (SDR) was used:

(4) $$SDR=\frac{k_{app}}{A}$$

The values of the specific degradation rate (SDR) for the HF 2-0 and HF 2-0.5 are given in Table 7. The higher value of SDR for HF 2-0.5 is in indication of the higher degradation rate of the membranes loaded with TiO_2_ nanoparticles.

Degradation of MB was also studied in synthetic seawater in order to prove the applicability of these systems in ultrafiltration as a pre-treatment step of reverse osmosis plants. Photocatalytic activity of TiO_2_ in a high concentration of chorine anions, was tested by repeating the MB degradation experiment with 25 g/L NaCl solution of dye (average salinity value of the Mediterranean Sea).

As shown in Figure 10, the addition of NaCl did not alter the degradation performance of HF 2-0.5 membranes towards MB under UV-A irradiation presenting a MB degradation of about 97% (after 300 min). An adverse effect of Cl^−^ anions has been found in some studies on the decolorization rate of commercial dyes using different oxidation process [67]. However, even if most of the oxidants were found to be sensitive to the presence of inorganic anions, TiO_2_ NPs were able to perform normally in the presence of NaCl.

A comparison between the results obtained in this work and those reported in the literature in terms of photocatalytic experiments, is provided in Table 8. It is difficult to identify which photocatalytically active membranes have exhibited the best performance due to the variation in multiple operational conditions, which strongly influence the entire membrane process (such as the content of titania nanoparticles, the type of light source and the target compound to degrade, as well as the membrane properties). However, the results obtained in this work are among the most promising. In fact, the photocatalytic degradation of MB registered in this work was higher than that reported by Almeida et al. [40] and Martins et al. [38] (Table 8).

## 4. Conclusions

TiO_2_ filled PVDF HFs were prepared by the phase inversion method. Two different approaches were investigated to improve the stability and the dispersion of TiO_2_ NPs in the dope solution using a chemical and a chemical/mechanical modification. The functionalized chemical/mechanical modified NPs showed the best performance in terms of long-term stability. The addition of the hydrophilic pore-former PEG in the dope solution enhanced TiO_2_ stabilization, and therefore, the long-term stability of the solution. Besides a better TiO_2_ dispersion in the membrane, an increase in water permeability was also obtained due to the combination of increased hydrophilicity and presence of a larger finger-like structure in the membrane due to PEG addition. Porous membranes with a good dispersion of the filler and with a pore size in the range of ultra/microfiltration were thus obtained. The addition of TiO_2_ improved the mechanical resistance of the produced HFs and increased the water permeability. Good catalytic activity, in spite of the low amount of catalysts added (0.5 wt %) was measured for the degradation of MB under UV-A irradiation (97% reduction). Degradation of MB was also studied in synthetic seawater degradation where the HFs confirmed their good catalytic performance as well (97% MB reduction).

## Figures and Tables

**Table 1:** Recent publications on PVDF-TiO_2_ membrane preparation.

TiO_2_ Type	Membrane Preparation and Particle Immobilization Technique	Main Results/Application	Reference
Nanoparticles on Membrane Surface			
85% anatase and 15% rutile TiO_2_ (20 nm, TitanPE Technologies)	NIPS+ (NPs in coagulation bath)	Improved membrane hydrophilicity and roughness. Superior retention properties (98.28%) of humic acid.	[18]
TiO_2_ NPs synthesized from Tetrabutyl titanate (TBOT)	Pre-treated PVDF film immersed in the TiO_2_ suspension	Improved membrane hydrophilicity and permeability, anti-fouling properties. Tests for adsorption of Cu^2+^ (removal of heavy metals via solid-phase extraction); decreased adsorption capacity of BSA.	[19]
---	Poly(acrylic acid) (PAA) plasma-grafted on commercial PVDF followed by dipping in aqueous TiO_2_ suspension.	Improved membrane hydrophilicity and permeability, anti-fouling properties. Tests for photodegradation of Reactive Black 5 (RB5) dye (wastewater treatment and re-use processes).	[20]
TiO_2_ NPs were synthesized from titanium (IV) iso-propoxide (TTIP)	Coating of TiO_2_ NPs onto PVDF membrane.	Super-hydrophobic PVDF membrane (for membrane distillation), improved rejection to NaCl and anti-fouling properties. Fouling tests with humic acid and CaCl_2_.	[21]
TiO_2_ (20 nm Degussa)	NIPS+ Coating of TiO_2_ NPs onto PVDF/SPES membrane.	Improved hydrophilicity, less tendency to fouling, improved BSA rejection. Membranes anti-bacterial properties tested on E. Coli via inhibition zone method.	[22]
85% anatase and 15% rutile TiO_2_ (20 nm, TitanPE Technologies)	NIPS+ (NPs in coagulation bath)	NPs had a significant effect on the membrane anti-fouling property. By increasing TiO_2_ content, membrane surface increased. Sufficient electrostatic repulsion appears between highly charged PVDF-TiO_2_ MMMs and HA aggregates, alleviating the adsorption phenomenon.	[23]
TiO_2_ NPs were synthesized from titanium (IV) iso-propoxide (TTIP)	NIPS+ A two-step modification methodology (polydopamine (pDA) coating method and vacuum filtration process)	Au-TiO_2_/pDA/PVDF nanocomposite membranes were tested for the degradation of tetracycline under visible light irradiation.	[24]
TiO_2_ NPs were synthesized from titanium (IV) iso-propoxide (TTIP)	Commercial PVDF membrane (Millipore Pty. Ltd.)	Laccase covalently immobilized on the TiO_2_ sol–gel coated PVDF membranes. Bio-catalytic membranes exhibited good Bisphenol A degradation efficiency over repeated use.	[25]
TiO_2_ (20 nm Degussa)	TiO_2_ coated on modified PAA-PVDF membrane	Reduced fouling tendency of PVDF membranes using whey solutions as foulant.	[10]
TiO_2_ NPs synthesized from Titanium tetrachloride	PVDF membrane coated by atomic layer deposition (ALD)	The deposition of TiO_2_ enhanced the hydrophilicity and fouling resistance of the PVDF membranes, which was more evident at higher ALD cycle numbers.	[11]
**Nanoparticles in Dope Solution**			
Anatase TiO_2_ (20 nm, Meidilin Nanometer Material)	TIPS	Microfiltration membranes showing uniform polymer spherulites, improved membrane performance.	[26]
Anatase TiO_2_ (20 nm, TitanPE Technologies)	NIPS	Improved membrane hydrophilicity and permeability, anti-fouling properties. Tests of photo-degradation of methylene blue (MB).	[27]
TiO_2_ (20 nm Degussa)	NIPS	Ultrafiltration membranes with improved membrane hydrophilicity and permeability, anti-fouling properties. Photodegradation experiments carried out on RB5; anti-fouling properties tested using BSA.	[28]
TiO_2_ (20–30 nm Degussa)	NIPS	Ultrafiltration membranes with good combination of flux and rejection and no particles aggregation. Foulants’ photocatalytic degradation was tested using Humic Acid (HA) (Natural organic matter, NOM, removal).	[29]
98% anatase TiO_2_ (20 nm Degussa)	NIPS	Membranes for treatment of colored wastewaters from textile or dye industry. Membrane wetting and dyes (Brilliant Green, BG, and Indigo Carmin, IC) photodegradation improved via ethanol membrane-pretreatment.	[30]
__	NIPS	PVDF–TiO_2_/PVDF dual layer hollow fiber membranes were prepared by the co-extrusion technique. The technique allows the nanoparticle distributed uniformly inside the membrane. The stability of dual layer hollow fiber membranes under UV changed in the surface during the whole operational period	[31]
TiO_2_ (20–30 nm Degussa)	NIPS	TiO_2_ NP improved the surface hydrophilicity and water permeation flux of the membrane. Anti-fouling properties tested using BSA	[32]
TiO_2_ Aeroxide P25 (85% anatase-15% rutile, ~20 nm)	NIPS	Under UV irradiation membrane super-hydrophilicity allowed to suppress pure water permeate flux decline and to reach higher fluxes. Fouled membranes after BSA filtration cleaned using water and UV irradiation. Permeate flux completely recovered after this cleaning.	[33]
TiO_2_ Aeroxide P25 (85% anatase-15% rutile, ~20 nm)	NIPS	Antibacterial activity against B. Subtilis which was enhanced by incorporating acid/alkali modified titania NPs into the polymer matrix.	[34]
TiO_2_ (20 nm)	TiO_2_/PVDF-HFP membranes prepared via electrospinning	The obtained membranes were tested in direct contact membrane distillation (DCMD), showing fluxes higher than those of commercial membranes.	[35]
TiO_2_ PC-101 (Japan Titan Kogyo, anatase type, 20 nm)	NIPS	Composite polymer electrolyte membranes exhibited excellent ionic conductivity, interfacial and electrochemical stability.	[36]
TiO_2_ synthesized in situ via Ti(OC_4_H_9_)_4_ hydrolysis	TiO_2_/PVDF-HFP membranes produced via NIPS	Enhanced porosity, ion conductivity; reduced activation energy for ion transport.	[37]
TiO_2_ (P25 EVONIK Industries)	TiO_2_/PVDF-TrFE and TiO_2_/zeolites (NaY)/PVDF-TrFE membranes prepared via Evaporation Induced Phase Separation (EIPS)	High membrane porosity which promoted MB degradation under UV light irradiation	[38]
TiO_2_ synthesized from tetrabutyl titanate (TBOT) by sol-gel method	TiO_2_/PVDF-TrFE membranes prepared via EIPS	The fabricated composite membranes manifested increased permittivity	[39]
TiO_2_ Aeroxide P25 (85% anatase-15% rutile, ~20 nm)	TiO_2_/PVDF-TrFE and TiO_2_/graphene oxide (GO)/PVDF-TrFE membranes produced by electrospinning	The presence of titania and GO improved the photocatalytic efficiency of the nanocomposite membranes towards the degradation of MB	[40]

**Table 2:** Composition of dope solution expressed (wt %).

Chemical	Type 1		Type 2	
	HF 1-0	HF 1-0.5	HF 2-0	HF 2-0.5
PVDF 6012	18	18	19	19
PVP k17	15	15	16	16
PEG400	-	-	10	10
H_2_O	5	5	-	-
NMP	62	61.5	55	54.5
TiO_2_	-	0.5	-	0.5

**Table 3:** Spinning parameters of the produced PVDF hollow fibers (HFs).

Spinning Condition	
Dope temperature	80 °C
Dope flow rate	11–12 g/min
Bore fluid composition and flow rate	NMP 30%; 13 g/min
Bore fluid temperature	50 °C
Outer coagulant	Tap water at room temperature
Air gap	24 cm
Spinneret dimension	O.D.–I.D. 1.6–0.6 mm

O.D.: outer diameter; I.D.: inner diameter.

**Table 4:** Detailed dimensions of the produced PVDF HFs.

Fiber	O.D. (mm)	I.D. (mm)	THICKNESS (mm)	POROSITY (%)
HF 1-0	1.85 ± 0.03	1.19 ± 0.02	0.33 ± 0.03	85.45 ± 0.18
HF 1-0.5	1.77 ± 0.01	1.37 ± 0.02	0.20 ± 0.02	84.99 ± 0.29
HF 2-0	2.22 ± 0.03	1.53 ± 0.02	0.34 ± 0.03	81.99 ± 1.88
HF 2-0.5	2.05 ± 0.12	1.55 ± 0.11	0.25 ± 0.16	83.06 ± 0.64

**Table 5:** Tensile properties of hollow fiber membranes produced in this work.

Fiber	Mechanical Tests	
	Young Modulus (N/mm^2^)	ε Break (%)
HF 1-0	41 ± 3	183 ± 15
HF 1-0.5	92 ± 2	168 ± 21
HF 2-0	54 ± 1	138 ± 8
HF 2-0.5	69 ± 1	169 ± 2

**Table 6:** Pore size data of produced HFs.

Measurement	HF 1-0	HF 1-0.5	HF 2-0	HF 2-0.5
Bubble point pressure (bar)	0.77 ± 0.1	0.83 ± 0.15	0.83 ± 0.18	0.90 ± 0.12
Smallest detected pore diameter (at 90% cff)	0.10 ± 0.02	0.10 ± 0.01	0.07 ± 0.01	0.09 ± 0.01
Mean flow pore diameter (μm)	0.13 ± 0.01	0.13 ± 0.03	0.14 ± 0.02	0.13 ± 0.03
Largest detected pore diameter (μm)	0.41 ± 0.08	0.55 ± 0.06	0.61 ± 0.07	0.51 ± 0.07

**Table 7:** Specific degradation rate of HF 2-0 and HF 2-0.5.

HF	Reaction Rate *k_app_* (min^−1^)	Area (m^2^)	SDR (m^2^∙min)^−1^
HF 2-0	0.0033	0.0036	0.916
HF 2-0.5	0.012	0.0036	3.33

**Table 8:** Comparison between the results obtained in this work and the literature in terms of photocatalytic activity.

Membrane Materials	Main Results/Application	Reference
PVDF-*g*-PAA/TiO_2_	TiO_2_-modified membranes assured a dye (RB5) removal in the range between 30% and 42% depending on titania concentration within 120 min UV lamp (254 nm, 15 W) operation	[20]
pDA/PVDF/Au-TiO_2_	Au-TiO_2_/pDA/PVDF nanocomposite membranes led to a degradation ratio of 92% of tetracycline within 120 min under visible light irradiation (300 W xenon light source placed at a distance of 10 cm and provided with a filter in order to block the light in a wavelength of less than 420 nm).	[24]
PVDF/TiO_2_	Membrane performance was evaluated in terms of pure water flux recovery under UV-A light (40 W, light intensity of 2.5 ± 0.2 mW/m^2^). The registered flux recovery ratios were about 100% within 60 min irradiation.	[27]
PVDF/TiO_2_	PVDF/TiO_2_ membranes allowed to achieve >99% of RB5 dye removal after 60 min irradiation (UV-C, 15 W).	[28]
PVDF/TiO_2_	HA-fouled membranes immerged in distilled water and irradiated with UV light (6 W, light intensity 0.04 mW/cm^2^). Water flux recovery increased by 11% when photocatalysis time was from 300 to 480 min.	[29]
PVDF/TiO_2_	BG photocatalytic degradation ratio was ~81% after 450 min UV light irradiation; IC photocatalytic degradation ratio was ~89% after 300 min UV light irradiation.	[30]
PVDF/TiO_2_	Pure water flux, measured during irradiation with a UV-lamp (9 W) reached 140 L/h m^2^ at 1 bar, preventing permeate flux decline observed in dark conditions.	[33]
PVDF-TrFE/TiO_2_	MB aqueous solution (10^−5^ M, Ph 6.8) was irradiated by means of 6 UV-A lamps (8 W, light intensity ~3.7 mW/cm^2^). The prepared TiO_2_/PVDF-TrFE membranes, containing 3 wt % of titania, led to a MB degradation of 77% after 90 min irradiation. TiO_2_/NaY/P(VDF–TrFE) membranes prepared with 8 wt % of titania and 8 wt % zeolites allowed to degrade 96% MB after 40 min irradiation.	[38]
PVDF-TrFE/TiO_2_ and PVDF-TrFE/TiO_2_/GO	P(VDF-TrFE)/TiO_2_ and P(VDF)-TrFE/TiO_2_/GO membranes (20 wt % titania in both membrane types) in contact with MB aqueous solution (10^−5^ M) were irradiated with a high-power UV-A LED source (light intensity 4 mW/cm^2^), leading to a MB removal of 92–93% within 110 min irradiation.	[40]
PVDF/TiO_2_	PVDF/TiO_2_ containing 0.5 wt % titania nanoparticles led to 97% MB degradation after 270 min irradiation with UV-A source (18 W, light intensity 2.7 mW/cm^2^).	*This work*
